# Effect of combination of three texture‐improving ingredients on textural properties of emulsified sausage‐containing salted egg white

**DOI:** 10.1002/fsn3.684

**Published:** 2018-06-04

**Authors:** Zhong Feng Wang, Tao Xu, Chu Yan Wang, Nan Deng

**Affiliations:** ^1^ Department of Biological and Environmental Engineering Hefei University Hefei China

**Keywords:** carrageenan, emulsified sausage, konjac flour, response surface methodology, salted egg, textural property

## Abstract

Response surface methodology based on Box–Behnken was used to assess the effects of three kinds of texture‐improving ingredients, namely, mixed starch (MS) (6%–8%) of sweet potato starch and glutinous rice flour, k‐carrageenan (CG) (0.4%–0.6%), and konjac flour (KF) (0.8%–1.2%), on the firmness, elasticity, and water holding capacity (WHC) of emulsified sausage (ES) made from pork and salted egg white (SEW). The three kinds of texture‐improving ingredients individually presented different effects on firmness, elasticity, and WHC. Their synergistic effects were significant. The three response models obtained by ANOVA were suitable to predict firmness, elasticity, and WHC. These models can also be used to design formulations for different types of sausage with different firmness and elasticity. The combination of MS (7.36%), CG (0.60%), and KF (1.20%) can produce SEW‐containing ES with remarkable firmness (224.04 g), elasticity (8.62), and WHC (8.41).

## 1 INTRODUCTION

Salted egg is largely popular in China and other Asian countries. Consumers prefer salted egg mainly for its yolk rather than salted egg white (SEW). The latter is too salty to be eaten directly because it contains 5%–10% or more NaCl, which results in large amount of protein resource waste. The SEW’s viscosity, thickness, and gelling were largely decreased by salting and SEW negatively affected the textural property in our previous experiment although egg white from fresh egg increased the hardness, chewiness, and penetration force of ES (Carballo, Barreto, & Colmenero, 1995).

Texture profile is one of the most important properties of ES, and it depends on the structure of matrix formed by proteins, water, and nonmeat ingredients. Starch (Carballo et al., 1995), carrageenan (Patrascu, Dobre, & Alexe, 2010), cellulose (Gibis, Schuh, & Weiss, 2015; Hu et al., 2016; Mara, Roger, Gizele, Queiroz, & Bastianello, 2014; Ruiz‐capillas, Triki, Herrero, Rodriguezsalas, & Jiménezcolmenero, 2012), konjac gel (Jiménez‐Colmenero, Triki, Herrero, Rodríguez‐Salas, & Ruiz‐Capillas, 2013; Triki, Herrero, Jiménezcolmenero, & Ruizcapillas, 2013), alginate (Kao & Lin, 2006), and other texture‐improving ingredients (TIIs) are widely used in meat products as potential fat substitutes to decrease fat content and improve textural properties. Starch is commonly used in sausage to improve texture, reduce cooking and purge losses, and increase hardness, chewiness, and penetration force (Carballo et al., 1995), and starchy grain from some plants also exhibits texture‐improving function (Fernández‐Diez et al., 2016). Carrageenan causes decreasing in emulsion stability, and increasing in water holding capacity, hardness, and cohesiveness of the formulated sausage samples (Ayadi, Kechaou, Makni, & Attia, 2009).

Among the above ingredients, Konjac flour (KF) is not only good for food texture but also for health function. The main composition of KF is glucomannan which is a neutral polysaccharide produced from the tuber of *Amorphophallus konjac* K. Koch (Lin & Huang, 2008). This polysaccharide performs many health functions, such as lowering serum cholesterol, low‐density lipoprotein cholesterol, and triacylglycerol (Arvill & Bodin, 1995). Therefore, KF is a good TII with health function. To improve the texture of SEW‐containing ES and increase the health function, based on our previous single factor experiment and simple combinatorial experiment, KF was used as a composition of ES. The k‐carrageenan (CG), starch from sweet potato, and flour from sticky rice were combined with KF in ES. The mathematical response models of each factor on firmness, elasticity, and water holding capacity (WHC) of ES were investigated. The present results can be used as a guide or reference in future studies or ES production.

## 2 MATERIALS AND METHODS

### 2.1 Material preparation

Fresh pork meat and fat were purchased from a local supermarket in Hefei, China. All visible fats and connective tissues were removed from the meat. Lean meat and fat were separately chopped into granulate in meat chopper. Uncooked salted egg was broken, and its egg white was separated from yolk. The chopped lean meat, fat, and egg white were separately packaged and stored at −20°C. For the konjac gel preparation, KF (Qiteng Trading Co., Ltd., Chengdu, China) and CG were mixed according to the required ratio. Water with a mass that is 20‐fold that of KF and CG mass was added to the mixture. Afterward, the mixture was kept at room temperature for approximately 15 min to allow the two ingredients to absorb water and swell. Subsequently, the mixture was cooled at 4°C. MS was prepared by mixing sweet potato starch with glutinous rice flour at the mass proportion of 1:1. Approximately, 0.4% of sodium carbonate on the bases of total mass of sausage was mixed with the MS.

### 2.2 ES preparation

On the basis of the designed scheme (Table 1), the frozen lean meat and fat granulate, and egg white were thawed at 4°C prior to sausage preparation. The lean meat granulate was first minced into like mud or paste. Then the starch, swelled KF and CG, and fat granulate were added and mixed with the lean meat mud. The mixed materials were further minced for about 1 min. Finally, the SEW and some flavoring ingredients, including sodium carbonate, were added and minced for 30 s. The batter was stuffed into collagen casings of 2 mm diameter. The sausage sections were vacuum packed and cooked for 40 min in water bath at 80°C and subsequently for 15 min at 90°C. These sections were cooled to ambient temperature in tap water after heating and stored at 4°C overnight for analysis the following day. The lean meat and fat contents in the sausage were at a proportion of 7:3.

**Table 1:** Box–Behnken design matrix and result data

Std No.	A (MS, %)	B (CG, %)	C (KF %)	Y_1_, g	Y_2_	Y_3_
1	−1 (6)	−1 (0.4)	0 (1.0)	150.99	6.89	7.05
2	1 (7)	−1	0	177.32	7.11	7.29
3	−1	1 (0.6)	0	199.21	7.79	7.34
4	1	1	0	200.00	7.88	7.62
5	−1	0 (0.5)	−1 (0.8)	132.68	6.42	6.88
6	1	0	−1	133.59	6.68	6.94
7	−1	0	1 (1.2)	159.75	8.01	8.42
8	1	0	1	187.45	8.19	8.89
9	0 (8)	−1	−1	143.13	6.23	6.24
10	0	1	−1	198.14	6.53	6.82
11	0	−1	1	211.21	6.96	8.25
12	0	1	1	224.46	8.65	8.35
13	0	0	0	169.00	8.41	7.59
14	0	0	0	161.25	8.19	7.66
15	0	0	0	169.82	8.48	7.56
16	0	0	0	166.13	8.12	7.71
17	0	0	0	168.75	8.21	7.58

*Note*. A: MS (mixed starch); B: CG (carrageenan); C: KF (konjac flour); Y_1_: Firmness; Y_2_: Elasticity; Y_3_: Water holding capacity.

### 2.3 Textural analysis and sensory evaluation

Sausage samples were cut into segments of 1 cm in height according to Lu, Luo, Li, & Li,^2014^); firmness of the samples was measured by P/50 probe of TA‐XT PLUS textural analyzer (SMS Co., UK). Each sample was compressed to half of the original height. Sausage elasticity was sensory evaluated by chewing and touching with fingers of 10 trained assessors. The sausages were cut into round pieces with about 5 mm thickness prior to elasticity evaluation. The assessors scored samples from 1 to 10, which corresponded to the lowest and highest elasticities, respectively.

### 2.4 WHC determination

The sausage was cut into round slices with 10 mm height. Each slice was weighed, placed on dried filter paper, and covered with the same paper. Approximately, 1 kg of sausage slices was placed on the top of paper for 10 min at room temperature. The sample was weighed when the weights and filters were removed. Evaluation was repeated three times for each sample. WHC was computed using Equation (1):

(1) $$\mathrm{WHC}=10\times (1-\frac{M_{0}-M_{1}}{M_{0}})$$

where *M*_0_ is the sample mass before pressing, and *M*_1_ is the sample mass after pressing.

### 2.5 Experimental design and statistical analysis

Response surface methodology (RSM) based on Box–Behnken in Design‐Expert 8.05 software (State‐Ease Inc.) was used to design the experimental scheme and assess the effects of MS (6%, 7%, 8%), CG (0.4%, 0.5%, 0.6%), and KF (0.8%, 1.0%, 1.2%) on the firmness, elasticity, and WHC of ES made from pork and SEW. A series of 17 individual experiments was conducted and result of the three textural properties of sausage was analyzed. The low, middle, and high levels of each variable factor are designated as −1, 0, and 1, respectively. These three variables and their respective ranges were selected on the basis of literature and supporting information from our preliminary experiments. The designed experimental scheme and result are shown in Table 1.

## 3 RESULTS AND DISCUSSION

### 3.1 Response models of experimental factors to textural properties

The designed experimental scheme and evaluated data of each point for firmness, elasticity, and WHC are shown in Table 1. Regression analysis was applied to fit the empirical model with the collected response variable data. The coefficients of the full regression model equation and their statistical significance were also determined and evaluated. After excluding insignificant term in relation to actual value for firmness (Y_1_), elasticity (Y_2_), and WHC (Y_3_), final models are given in Equations (2), (4):

(2) $$\begin{array}{cc} Y_{1}= & 166.62+6.97A+17.52B+21.92C-6.39\mathrm{AB}\\ & +6.70\mathrm{AC}-14.44\mathrm{BC}-12.99A^{2}+27.88B^{2}; \end{array}$$

(3) $$Y_{2}=8.28+0.46B+0.74C+0.35\mathrm{BC}-0.32A^{2}-0.55B^{2}-0.64C^{2};$$

(4) $$Y_{3}=7.62+0.13A+0.12B+0.90C+0.07\mathrm{AC}-0.12\mathrm{BC}-0.33B^{2}+0.14C^{2};$$

where *A*, B, and C were coded terms for the three TIIs that were selected, that is, MS, CG, and KF, respectively. A positive sign in front of the terms indicated synergistic effect, whereas negative sign indicated antagonistic effect. The obtained results were analyzed by ANOVA to assess the goodness of fit.

### 3.2 ANOVA

According to the ANOVA results shown in Table 2, the *F* values were sufficiently high. Furthermore, the values of Prob > *F* less than 0.05 indicated that the model terms were significant. The lack of fit values of the three models was not significantly relative to the pure error. The goodness of fit of the models was evaluated by the determination coefficient (*R* ^2^), adjusted determination coefficient (Adj *R* ^2^), and predicted determination coefficient (Pred *R* ^2^, data not shown). High *R* ^2^, Adj *R* ^*2*^, and Pred *R* ^*2*^ for all the analyzed properties (Table 2) also revealed that the models were statistically significant. All adequate precision values were higher than 4, which indicated adequate signals. These models could be used to navigate the design space.

**Table 2:** ANOVA table for responses

Source	Y_1_ (Firmness)		Y_2_ (Elasticity)		Y_3_ (Water holding compacity)	
	*F* value	Prob > *F*	*F* value	Prob > *F*	*F* value	Prob > *F*
Model	120.56	<0.0001	68.68	<0.0001	278.42	<0.0001
A	37.40	0.0005	4.16	0.0806	48.93	0.0002
B	233.2	<0.0001	99.16	<0.0001	39.38	0.0004
C	370.18	<0.0001	262.06	<0.0001	2209.24	<0.0001
AB	15.71	0.0054	0.25	0.6323	0.69	0.4328
AC	17.29	0.0043	0.10	0.7672	6.70	0.0360
BC	42.00	0.0003	28.60	0.0011	20.52	0.0027
A^2^	68.43	<0.0001	24.90	0.0016	2.30	0.1729
B^2^	315.26	<0.0001	75.02	<0.0001	159.15	<0.0001
C^2^	0.16	0.6985	102.45	<0.0001	26.23	0.0014
Lack of fit	0.65	0.6229	0.32	0.8136	0.39	0.7646
*R* ^*2*^	0.9936		0.9888		0.9972	
Adj *R* ^:^	0.9853		0.9744		0.9936	
C.V.%	1. 85		1.72		0.72	

*Note*. A: MS (mixed starch); B: CG (carrageenan); C: KF (konjac flour).

**Figure 1:**
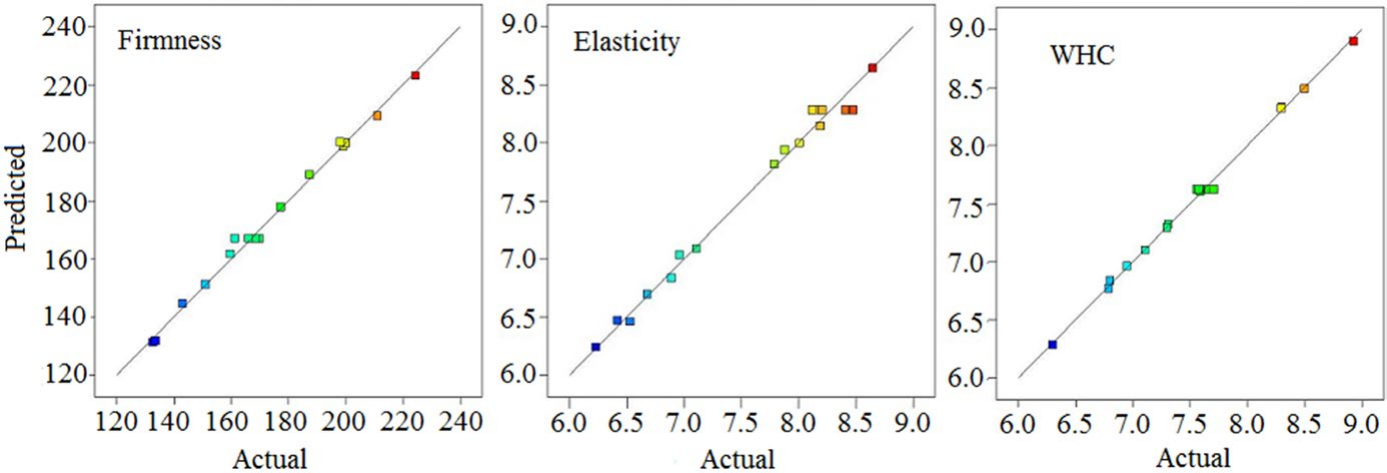
Diagnostic plots for model adequacy of firmness, elasticity, and WHC

The significant model terms for firmness were A, B, C, AB, AC, BC, A^2^, and B^2^. Among them, AB, BC, and A^2^ presented a negative effect on firmness. The linear term of C (KF) and its quadratic and linear term of B (CG) with *F* values of 370.18, 315.26, and 233.20, respectively, showed the most remarkable effect on firmness sequentially. The *F* values for other significant terms were less than those of these three former terms. Furthermore, significant model terms for elasticity were B, C, BC, A^2^, B^2^, and C^2^. Among them, the linear term of C and its quadratic term with *F* values of 262.06 and 102.45, respectively, displayed the most remarkable effect consecutively. By contrast, all the quadratic terms presented a negative effect on elasticity. Additionally, the significant model terms for WHC were A, B, C, AC, BC, B^2^, and C^2^. The linear term of C exhibited the highest effect on WHC with *F* value of 2209.24, which was about 14 times more than that of the second significant term, that is, quadratic term of B with 159.15. The linear term of interaction between B and C along with the quadratic term of B presented negative effect on WHC. The predicted values according to the models and corresponding to the actual value of each textural property are presented in Figure 1. Both the figures in Figure 1 and the correlation coefficients in Table 2 showed a high consistency and correlation of predicted values from the three models with actual values from the experiment.

### 3.3 Interaction effect of tiis on the three textural properties

**Figure 2:**
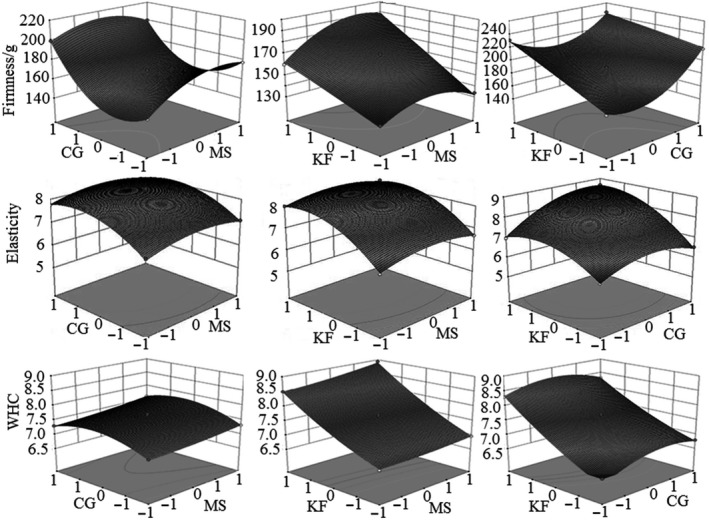
Response surface for firmness, elasticity, and WHC (water holding capacity) to MS (mixed starch), CG (carrageenan), and KF (konjac flour)

The effect of interaction between any two of the three factors on each textural property could be observed in the 3D response surface plots generated by RSM and are shown in Figure 2. Evidently, the firmness increased with the increasing in CG and KF amount (Figure 2; firmness). The interaction effect between MS and KF on firmness was positive, whereas the interaction effect between MS and CG or CG and KF produced negative effect. The most remarkable firmness (224.46 g) was observed at the combination of MS of 7.0%, CG of 6.0%, and KF of 1.2% (Table 1). The same factor condition also produced the highest elasticity (8.65) (Table 1). However, only the interaction between CG and KF showed significant positive effect on elasticity (Figure 2; elasticity—right figure). The good effect of interaction between MS and KF on WHC could be observed in Figure 2; WHC—middle figure). The negative interaction effect between CG and KF on WHC also existed (Figure 2; WHC—right figure). The highest WHC (8.93) was observed at MS of 8.0%, CG of 0.5%, and KF of 1.2% (Table 1).

### 3.4 Optimization and verification of results from models

The optimum conditions for the three variables, namely, MS, CG, and KF, were obtained using the numerical optimization feature of Design‐Expert 8.05 software. The software searched for a combination of factors that simultaneously satisfy the requirements placed on each of the responses and factors. All the factors and the responses with high‐ and low‐limit experimental regions should satisfy the creations defined for the optimum working condition. The firmness, elasticity, and WHC should also be maximized.

The predicted optimum properties were firmness of 224.04 g, elasticity of 8.62, and WHC of 8.41 at the optimized condition of MS (7.36%), CG (0.60%), and KF (1.20%). The firmness, elasticity, and WHC values from the verification experiment were highly in agreement with the results predicted from the regression models with small errors of 2.80%, 2.67%, and 2.02%, respectively. These results verified the validity of the models and the existence of an optimal point. The constraints for optimization, optimum conditions, and predicted and experimental results are listed in Table 3.

**Table 3:** Models verification based on optimum conditions

Factors	Goal	Constraints				Importance
		Lower limit	Upper limit	Lower weight	Upper weight	
MS	In range	6.00	8.00	1	1	3
CG	In range	0.40	0.60	1	1	3
KF	In range	0.80	1.20	1	1	3
*F*	Maximize	132.68	224.46	1	1	4
E	Maximize	6.23	8.65	1	1	5
WHC	Maximize	6.30	8.93	1	1	4
		Texture	*F*	E	WHC	D
Optimum conditions	MS = 7.36	Predicted	224.04	8.62	8.41	0.93
	CG = 0.60	Experimental	230.41	8.85	8.24	
	KF = 1.2	Percentage error	2.80	2.67	2.02	

*Note*. A: MS (mixed starch); B: CG (carrageenan); C: KF (konjac flour); *F*: firmness; E: elasticity; D: Desirability; WHC: water holding capacity.

## 4 DISCUSSION

Starch is commonly used in sausage to improve texture, reduce cooking, and purge losses, and increase hardness, chewiness, and penetration force (Carballo et al., 1995). In our study, the positive effect of MS on firmness, elasticity, and WHC was lower than that of CG and KF. MS was the mixture of starch from sweet potato and flour from sticky rice. Starchy grain from some plants exhibits texture‐improving function (Fernández‐Diez et al., 2016). In our preliminary experiment, MS displayed better effect on the texture of this kind of sausage than that of only starch used, especially on WHC; the difference could be due to the function of amylopectin in sticky rice. Carrageenan decreased the emulsion stability and increased the WHC, hardness, and cohesiveness of formulated sausage samples.

Mixed gel of 1% konjac and 0.25% gellan gum was used in fat‐reduced frankfurters to increase firmness (Lin & Huang, 2003). An increase amount of konjac gel in fat‐reduced dry fermented sausages increased the hardness and chewiness and decreased the cohesiveness; nevertheless, no clear effect was observed on springiness (Ruiz‐capillas et al., 2012). The present study result was consistent with that of previous report about the effect of konjac gel on increasing firmness and decreasing cohesiveness of sausages. The difference could be possibly attributed to the synergistic effect of the three TIIs, especially the synergistic effect of KF with CG.

The gel formation of KF proceeds in the pH range of 9–10, and gels are thermally stable at temperatures higher than 200°C (Huang & Lin, 2010). According to our previous experiment (data not shown), good effect of KF in ES was based on its combined use with sodium carbonate which could increase the pH value. The KF‐containing sausage without sodium carbonate was soft and adhesive. However, the high pH value of product will produce alkaline flavor which is not good for consumer’s acceptance. In the present study, about 0.4% sodium carbonate was employed to the ES. More sodium carbonate would increase alkaline taste of product. Therefore, the sodium carbonate should be used as less as possible, if only the texture is satisfactory.

## 5 CONCLUSION

This study demonstrated that the combination of MS, CG, and KF could improve the textural characteristics of SEW‐containing ES. The three kinds of used TIIs individually presented different effects on firmness, elasticity, and WHC, and their synergetic effects were significant. The three response models obtained from Design‐Expert 8.05 were suitable to predict firmness, elasticity, and WHC at a given condition. These models can be used to design the formulation of TIIs for different types of sausage with different firmness and elasticity values.

## CONFLICT OF INTEREST

None declared.

## ETHICAL STATEMENT

There are not any human or animal testing involved in this study.
